# Laparoscopic Reduction of Intussusception in Children in Restricted Environment: Our Initial Experience Supports Timely Resort to Minimally Invasive Surgery

**DOI:** 10.4274/balkanmedj.2018.0627

**Published:** 2019-01-01

**Authors:** Jakov Mihanović, Robert Karlo, Ivan Bačić, Nataša Skitarelić

**Affiliations:** 1 Department of Surgery, Zadar General Hospital, Zadar, Croatia; 2 Department for Health Studies, University of Zadar, Zadar, Croatia; 3 Department of Pediatrics, Zadar General Hospital, Zadar, Croatia

To the Editor,

Intussusception is a true pediatric emergency requiring a multidisciplinary approach. Clinical suspicion should lead directly toward an abdominal ultrasound or a contrast enema. As a part of modern point-of-care management, ultrasound has emerged as a readily available diagnostic modality even in rural hospitals. After establishing a diagnosis, the pediatric or general surgeon should decide upon further treatment, preferably enema reduction (1). The drawbacks of conservative treatment are reduction failure in 20% of the cases, risk of perforation, radiation load, higher rate of recurrence than that with operative treatment, and oversight of the potential leading point. Laparoscopic-assisted hydrostatic reduction might play a role in nonreducible cases (2). Standard surgery offers least recurrence rate along with an option for immediate resection in cases of perforation and bowel necrosis or when the leading point is identified. Removal of the appendix vermiformis is at the surgeon’s discretion. The disadvantages are the need for general anesthesia, potential iatrogenic injuries, and postoperative complications such as bowel obstruction, adhesions, surgical site infection, and trocar site hernia (3). The hospital type and volume have an influence on the treatment algorithm as demonstrated by Jen and Shew (4). Patients with intussusception had less chance for undergoing operation when treated at a children’s tertiary hospital compared to that in general or non-children hospitals. The median number of cases in the children’s hospital in their study was 2.5 cases per year (range 1-5 cases per year), which correlates with the incidence of children operated for intussusception in our general hospital (10 cases in 5 years). The incentive for this letter was the gap observed between the current guidelines and the reality. Much of the delay and stress in diagnosing and treating intussusception could be bypassed with early ultrasound examination followed by expedite minimally invasive surgical treatment. Ultrasound-guided contrast enema study in our facility is generally available only during working hours. Paradoxically, laparoscopic equipment is accessible to our patients even in an emergency setting. Modern surgical treatment of intussusception favors laparoscopic reduction when perforation and shock are ruled out (3,5). Apelt et al. (3) conducted a systematic review in 2012 and concluded that tertiary centers with adequate minimally invasive skills should establish laparoscopy as the primary surgical technique. It is reasonable to expand this assertion to all hospitals with appropriate laparoscopic expertise. The transport to a tertiary center in our circumstances implies the injudicious delay, and therefore, it is not recommended.

After having treated two children with clinical and ultrasound signs of intussusception in the previous year, we commend the laparoscopic approach. The abdominal cavity was explored through a 5-mm endoscope with two additional ports of 5 and 10 mm in the suprapubic and left lower quadrant as done for standard laparoscopic appendectomy. The reduction of intussusception with addition of appendectomy was successfully performed in 21- and 31-month-old boys, followed by swift and uneventful recovery. Written informed consent was obtained from the parents of the operated children.

The treatment of children with intussusception in suboptimal conditions as found in hospitals without facilities for therapeutic enema supported with our initial successful experience indicates that a shift toward early minimally invasive surgery should be considered (Figure 1).

## Figures and Tables

**Figure 1:**
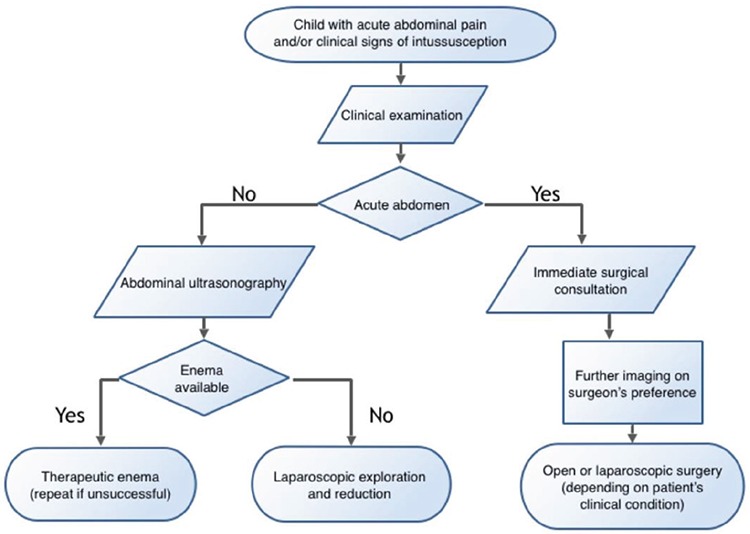
Proposed flowchart for children with symptoms of intussusception in hospitals with limited facilities.

## References

[1] Ito Y, Kusakawa I, Murata Y, Ukiyama E, Kawase H, Kamagata S, et al (2012). Japanese guidelines for the management of intussusception in children, 2011. Pediatr Int.

[2] Geltzeiler CB, Sims TL, Zigman AF (2015). LAHRI: Laparoscopic-Assisted Hydrostatic Reduction of Intussusception. J Laparoendosc Adv Surg Tech A.

[3] Apelt N, Featherstone N, Giuliani S (2013). Laparoscopic treatment of intussusception in children: a systematic review. J Pediatr Surg.

[4] Jen HC, Shew SB (2009). The impact of hospital type and experience on the operative utilization in pediatric intussusception: a nationwide study. J Pediatr Surg.

[5] Bonnard A, Demarche M, Dimitriu C, Podevin G, Varlet F, François M, et al (2008). Indications for laparoscopy in the management of intussusception: A multicenter retrospective study conducted by the French Study Group for Pediatric Laparoscopy (GECI). J Pediatr Surg.

